# Complete chloroplast genome of *Senna spectabilis* (DC.) H.S. Irwin & Barneby (Fabaceae) and phylogenetic analysis

**DOI:** 10.1080/23802359.2020.1790323

**Published:** 2020-07-15

**Authors:** Zhaowan Shi, Guozheng Shi, Kunkun Zhao, Bing Sun

**Affiliations:** a Research Institute of Tropical Forestry, Chinese Academy of Forestry, Guangzhou, China; b Experimental Station of Research Institute of Tropical Forestry, Chinese Academy of Forestry, Ledong, China

**Keywords:** *Senna spectabilis*, chloroplast genome, ornamental tree, phylogenetic analysis

## Abstract

*Senna spectabilis* (DC.) H.S. Irwin & Barneby is a popular ornamental tree as well as a traditional medical plant in Cameroon. In this study, we sequenced and annotated the complete chloroplast genome of *S. spectabilis* and reconstructed the phylogenetic relationship of the tribe Cassieae. The length of the chloroplast genome was determined to be 162,754 bp, containing a pair of inverted repeats of 25,413 bp which separated by a small single-copy (SSC) region of 20,161 bp and a large single-copy (LSC) region of 91,767 bp. The cp genome encodes 128 genes, including 83 protein-coding genes, 37 tRNA genes, and eight rRNA genes. The percentage of total GC content of this genome was 35.7%. The phylogenetic analysis indicates that *S. spectabilis* with the sampled *Senna* species formed a well-supported monophyletic clade.

*Senna spectabilis* (DC.) H.S. Irwin & Barneby is a popular ornamental tree with bright yellow and fragrant flowers, which is native to tropical America and widely cultivated in south Yunnan and Guangdong Province of China (Chen et al. 2010). Moreover, *S. spectabilis* was also used as medical plant in Cameroon to treat anxiety, constipation, epilepsy, and insomnia (Nkantchoua et al. 2018). The leaves, flowers, and fruits of *S. spectabilis* are important source of cytotoxic alkaloids (de Albuquerque Melo et al. 2014). However, the phylogenetic relationship of the genus *Senna* and the phylogenetic position of *S. spectabilis* are still unresolved. In the present study, we report the first complete chloroplast genome of *S. spectabilis* and perform the phylogenetic analysis of the tribe Cassieae employ chloroplast phylogenomics.

Total genomic DNA was isolated from ca. 15 mg silica gel dried leaves following the modified CTAB method (Doyle and Doyle 1987). Leaf sample of *S. spectabilis* was collected from a cultivated tree in Experimental Station of Research Institute of Tropical Forestry, Chinese Academy of Forestry, Ledong County (18°41.651′N, 108°47.135′E), Hainan Province, China. The voucher specimens (accession number: S200009) were deposited at the herbarium of South China Botanical Garden (IBSC). Paired-end reads (PE = 150 bp) were produced using a BGISEQ-500 platform at Beijing Genomics Institute (Shenzhen, China). Before assembly, the raw reads were filtered by the program Trimmomatic v.0.33 (Bolger et al. 2014) with default parameters. Then the trimmed reads were assembled into complete chloroplast genome by using NOVOPlasty 2.6.3 (Dierckxsens et al. 2017) with kmer length set to 59 base pairs. Annotation of the cp genome was performed on Geneious version 11.0.3 (Kearse et al. 2012), while the tRNA genes were annotated on ARAGORN (Laslett and Canback 2004). The annotated plastome of *S. obtusifolia* (GenBank accession number: MK817504) was used as a reference for assembly and annotation.

The complete cp genome of *S. spectabilis* (GenBank accession number: MT635191) was 162,754 bp in length, containing a small single-copy region (SSC) of 20,161 bp, a large single-copy region (LSC) of 91,767 bp and a pair of inverted repeats (IRs) of 25,413 bp. The GC contents of this genome is 35.7%, while the GC contents of LSC, SSC, and IRs are 33.1%, 29.3%, and 42.9%, respectively. A total of 128 unique genes were identified from this genome, including 83 protein-coding genes, 37 tRNA genes, and eight rRNA genes. Among these genes, 15 genes (*trnK*-*UUU*, *rps16*, *trnG*-*UCC*, *atpF*, *rpoC1*, *trnL*-*UAA*, *trnV*-*UAC*, *petB*, *petD*, *rpl16*, *rpl2*, *ndhB*, *trnI*-*GAU*, *trnA*-*UGC*, and *ndhA*) have one intron and three genes (*clpP*, *rps12*, and *ycf3*) have two introns.

To identify the phylogenetic position of *S. spectabilis*, we reconstructed the phylogeny of the tribe Cassieae. The matrix includes all of the reported plastomes of this tribe and an outgroup from the genus *Libidibia*. The plastomes of the 14 accessions were aligned using MAFFT (Katoh and Standley 2013). Then the phylogenetic analysis was performed by using RAxML (Stamatakis 2006) with 1000 bootstrap replicates. The results support the monophyly of the genus *Senna* (Figure 1). *Senna spectabilis* is sister to the clade which including *S. obtusifolia*, *S. tora* and *S. occidentalis*.

**Figure 1.**
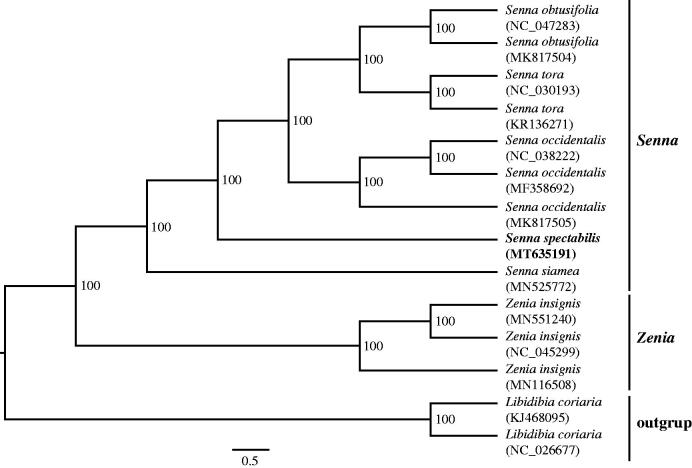
Maximum-likelihood tree based on 12 complete chloroplast genomes of Cassieae. *Libidibia coriaria* was used as outgroup. Bootstrap support values are shown at the branches.

## Data Availability

The data that support the findings of this study are openly available in GenBank of NCBI at https://www.ncbi.nlm.nih.gov, reference number MT635191.
